# Toll-Like Receptor 9 in Breast Cancer

**DOI:** 10.3389/fimmu.2014.00330

**Published:** 2014-07-22

**Authors:** Jouko Sandholm, Katri S. Selander

**Affiliations:** ^1^Turku Centre for Biotechnology, University of Turku and Åbo Akademi University, Turku, Finland; ^2^Department of Pathology, Lapland Central Hospital, Rovaniemi, Finland; ^3^Division of Hematology-Oncology, Department of Medicine, University of Alabama at Birmingham, Birmingham, AL, USA; ^4^Comprehensive Cancer Center, University of Alabama at Birmingham, Birmingham, AL, USA

**Keywords:** TLR9, breast cancer, invasion, inflammation, prognosis

## Abstract

Toll-like receptor 9 (TLR9) is a cellular DNA receptor of the innate immune system. DNA recognition via TLR9 results in an inflammatory reaction, which eventually also activates a Th1-biased adaptive immune attack. In addition to cells of the immune system, TLR9 mRNA and protein are also widely expressed in breast cancer cell lines and in clinical breast cancer specimens. Although synthetic TLR9-ligands induce cancer cell invasion *in vitro*, the role of TLR9 in cancer pathophysiology has remained unclear. In the studies conducted so far, tumor TLR9 expression has been shown to have prognostic significance only in patients that have triple-negative breast cancer (TNBC). Specifically, high tumor TLR9 expression predicts good prognosis among TNBC patients. Pre-clinical studies suggest that TLR9 expression may affect tumor immunophenotype and contribute to the immunogenic benefit of chemotherapy. In this review, we discuss the possible contribution of tumor TLR9 to the pathogenesis and treatment responses in breast cancer.

## Introduction

Toll-like receptor 9 (TLR9) is a DNA receptor that recognizes microbial and vertebrate DNA (1–5). Initially, TLR9 was thought to recognize specifically the CpG sequence in DNA (1, 6). The sequence-requirement may, however, be relevant only for the synthetic, oligonucleotide TLR9-ligands in the phosphorothioate backbone, and also CpG sequence-independent TLR9 activation by DNA has been reported (6–8). Like the other TLRs that recognize nucleic acids (TLR3, TLRs 7–8, and TLR13), TLR9 is located at the endoplasmic reticulum in resting cells (9, 10). When DNA enters the cell, TLR9 translocates to the endosomal/lysosomal compartment where ligand recognition and binding takes place (9, 11). DNA recognition by TLR9 initiates a downstream signaling cascade, which includes the adaptor molecule MyD88 (12, 13). As an effector of the innate immune system, stimulation of TLR9 induces a NF-κB-mediated rapid inflammation, characterized by increased expression of various interleukins and cytokines. A common feature for the nucleotide-sensing TLRs is the induction of both antiviral and antitumoral type I interferons (IFNs) from plasmocytoid dendritic cells (pDCs) (14). Eventually, this inflammation also activates the adaptive immune system, which then results in the clearance of the invading pathogens and the infected cells (2, 15). A similar inflammatory response, mediated via TLRs, also takes place during sterile tissue damage (16–19). In addition to DNA, other biological molecules have also been suggested to induce TLR9-mediated responses. Such molecules include the malaria pigment hemozoin and histone proteins (17, 20–22). TLR9 was recently shown also to recognize RNA–DNA hybrids (23).

## TLR9 Expression in Breast Cancer

Toll-like receptor 9 expression has been detected in cells of breast milk (TLR9 mRNA) and also in normal epithelial cells of the mammary gland (TLR9 protein) (24, 25). TLR9 mRNA and protein are also widely expressed in various human cancer-cell lines as well as in clinical cancer specimens, including breast, prostate, brain, gastric, renal cell carcinoma, and esophageal tumors (24, 26–33). Specifically in breast cancer, TLR9 protein expression has been detected both in the epithelial cancer cells as well as in the fibroblast-like cells associated with the tumors (24, 26, 29, 34). Consistent with the endosomal/lysosomal localization of TLR9 at the subcellular level, in breast cancer cells *in vitro*, TLR9 appeared punctate in intracellular fluorescence staining, located especially in the perinuclear region, where these organelles are located (35). Of the five human TLR9 isoforms (A–E), mRNA expression of the TLR9 A and B isoforms has been studied and detected in breast cancer specimens (36, 37).

## TLR9 as a Prognostic Factor in Breast Cancer

The prognostic significance of TLR9 in cancers appears to be bimodal. In some cancers, such as glioma, prostate cancer, and esophageal adenocarcinoma, high tumor TLR9 expression has been associated with poor survival whereas in others, such as triple-negative breast cancer (TNBC) or renal cell carcinoma, low tumor TLR9 expression upon diagnosis predicts poor prognosis (27, 30, 32, 33, 38, 39). We demonstrated recently that although widely expressed in all clinical subtypes of breast cancer, TLR9 expression has significant, prognostic significance only in TNBC that lack the expression of estrogen (ER), progesterone (PR), and HER2 receptors. More specifically, low tumor TLR9 expression upon diagnosis was associated with a significantly shortened disease-free-specific survival (29, 32). Furthermore, although we demonstrated that low-TLR9–TNBC cells become highly invasive in hypoxic conditions, it is currently unclear whether this mechanism contributes to the poor survival of the breast cancer patients that have hypoxic, low-TLR9–TNBC tumors. The mechanism for the increased invasion in hypoxia when TLR9 is absent is also not known (32). In addition to the actual tumor cells, the TLR9 expression status of tumor-associated fibroblast-like cells has also been shown to be of prognostic value in breast cancer. In this context, high TLR9 expression was associated with better prognosis (34). This study did not, however, assess triple-negative status of the cancers, and the exclusion of metastatic and neoadjuvant-treated patients probably counter selected against patients of the TNBC subtype.

## Effects of TLR9 Stimulation on Cellular Invasion

Synthetic TLR9-ligands, the CpG sequence-containing oligonucleotides (CpG–ODNs, such as ODN M362) that mimic bacterial DNA, are strong inducers of inflammation in cells of the immune system (40, 41). These oligonucleotides mimic bacterial DNA based on their high CpG content and unmethylated cytosines. CpG–ODNs are taken up into cells via DEC-205, a multilectin cell surface receptor, which is expressed in various cell types (42). These same compounds induce cellular invasion in macrophages, mesenchymal stem cells, and in cancer cells of various origins *in vitro* (24, 28, 43, 44). In breast cancer cells, such synthetic TLR9 ligand-induced invasion has been detected both in ER-positive and ER-negative breast cancer cells (24, 28, 35). This invasive effect is mediated via TLR9, and it is blocked by chloroquine, an inhibitor of endosomal acidification and an inhibitor of TLR9 signaling. Downstream of TLR9, such invasion is mediated via TRAF6, but not MyD88 (24, 28, 35). At the proteolytic level, CpG–ODN-induced invasion is associated with down-regulation of tissue inhibitor of matrix metalloproteinases-3 (TIMP-3) and activation of matrix metalloproteinase-13 (MMP-13) (24, 28, 35, 44). Interestingly, although methylation of cytosines in CpGs has been shown to decrease their pro-inflammatory effects, the invasive effects of these molecules are independent of their methylation status (35, 40, 45). CpG–ODNs can form various secondary structures, including homopolymer duplexes and hairpins, containing stem loop structures. The stem loop secondary structure appears important for the invasive effects of the CpG–ODN (35). Furthermore, the invasive effects can also be seen with non-CpG sequence-containing ODNs that in inflammatory experiments act as TLR9 antagonists (24, 46). The synthetic, phosphorothioate-backbone-modified CpG–ODNs do not exist in nature. Thus, for this invasion to have physiological significance, it would have to be caused also by natural DNA in the phosphodiester backbone. In prostate cancer cell lines and in gastrointestinal cancer cell lines, bacterial DNA (purified from *Escherichia coli* or *Helicobacter pylori*, respectively) also has similar, stimulatory effects on invasion (28, 43). Whether microbe-derived DNA similarly induces invasion in breast cancer cells is not known. We, however, demonstrated recently that self-DNA, which is derived from chemotherapy-treated, dead cancer cells is rapidly taken up into surviving cancer cells, where it serves as an invasion-inducing TLR9 ligand (47). This cellular uptake is possibly endocytosis or pinocytosis-mediated, since fluorescently labeled, dead cancer-cell-derived DNA, which was added to cell culture medium, was seen inside the recipient cells rapidly, within 15 min. However, similar to other reported TLR9-mediated effects of cell-derived self-DNA, complex formation of such cell-derived DNAs with the cationic antimicrobial peptide LL-37 enhanced DNA uptake into viable breast cancer cells, and was a requirement for the invasion-inducing effects (47, 48). This scenario may be physiologically relevant since LL-37 is expressed also in breast cancers (49, 50). Interestingly, the effects of cell-DNA on invasion are mediated via cathepsins and surprisingly, not via MMPs, which are the mediators for CpG–ODN-induced invasion (44, 47, 51, 52). DNA that was derived from intact, proliferating cancer cells did not induce invasion. This suggests that the invasive effect requires a certain DNA-structure, either alone or in complex with LL-37. Such DNA-structures could possibly be formed upon DNA degradation by nucleases. Whether self-DNA-induced and TLR9-mediated cancer cell invasion takes place *in vivo* in breast or any cancer is currently unknown. In principle, however, such DNA-induced and TLR9-mediated cancer cell invasion could represent a novel mechanism of treatment resistance. Since tumor growth is the sum of local proliferation and local invasion, such treatment resistance could theoretically manifest as no change or even increase in tumor size despite treatment. Finally, TLR9 appears to have also ligand-independent invasive activity. Down-regulation of TLR9 in MDA-MB-231 breast cancer cells through siRNA results in decreased *in vitro* invasion in the absence of exogenous DNA. The decreased invasion of the TLR9 siRNA cells was associated with decreased MMP activity and increased expression of TIMP-3 (32). Similar effects were also detected by TLR9 siRNA in brain cancer cells *in vitro* (53). These TLR9 expression-induced changes in the cellular invasive machinery suggest that TLR9, as a DNA-binding protein, might also have effects on gene transcription. TLR9 expression has indeed been detected in the nuclei of renal cell carcinoma tumor samples (30), but whether or not it can directly affect gene expression, requires further experimenting.

## Effects of TLR9 Stimulation on Inflammation

Toll-like receptor 9 agonists have various well documented pro-inflammatory effects in cells of the immune system (40, 41, 48, 54). Whether synthetic TLR9 agonists also induce the expression of inflammatory mediators in breast cancer cells, is not known. In cells of the immune system, a key characteristic of the TLR9-induced innate immune response is the promotion of a strong type I T helper cell (Th1) adaptive immune response. This includes both CD8^+^ T-cell responses and antigen-specific antibody responses (55). Since CD8^+^ T-cells are capable of immunologic tumor cell destruction, CpG–ODNs have been tested both as monotherapy and as an adjuvant for cancer vaccines, against various cancer types in pre-clinical cancer models, including breast cancer (55). In mouse models of breast cancer, CpG–ODN treatment resulted in the eradication of orthotopic tumors (56, 57). CpG–ODN treatment also induced an immunologic memory against tumor challenge, which was associated with an up-regulation of IFN-γ-positive CD4^+^ and CD8^+^ T-cells (56, 57). CpGs, when given as an adjuvant with a peptide vaccine, also prevented the formation of spontaneous tumors in a mouse model of HER2-positive breast cancer (58). Although the direct growth inhibitory effects of CpG–ODNs on cancer cells are quite weak *in vitro*, certain modifications in the CpG structure have resulted in increased tumor growth inhibition, also in nude mouse models *in vivo*, suggesting direct tumor effects of these compounds (24, 59–61). Furthermore, when given in a combination, the immunomodulatory ODN was also shown to potentiate the efficacy of trastuzumab, an anti-HER2-antibody, in a mouse model of breast cancer (59). In conclusion, these pre-clinical experiments suggest that TLR9 ligands can directly inhibit the growth of breast cancer cells *in vitro* and *in vivo*, and they can enhance anti-tumor immunity, possibly via inducing a Th1 adaptive immune response. These studies have not, however, addressed the role of TLR9 expression in tumors vs. host in these responses. Despite the successful pre-clinical results, CpG treatment has demonstrated anti-tumor activity only in select patients in clinical trials. There are, however, no reports on their efficacy in breast cancer trials (55). Finally, the discrepancies between the *in vitro*-observed, unwanted tumor invasion-promoting effects and the favorable, most likely immune system-mediated anti-tumor effects of the synthetic TLR9-ligands are likely explained by the pharmacokinetics of these compounds. After s.c. and i.v. administration, highest concentrations of TLR9 ligands are detected in plasma, kidneys, and organs of the reticuloendothelial system, and much less so in tumor tissues (59).

Self-DNA has been shown to have TLR9-mediated inflammatory effects in other cell types, especially when complexed with LL-37, which is expressed in various tissues (16, 48, 52, 62). We demonstrated recently that self-DNA, which is derived from doxorubicin-killed breast cancer cells, induces mRNA expression of various inflammatory mediators in living, TLR9-expressing cells. Furthermore, while assessing treatment responses to doxorubicin in a mouse model of TNBC, we discovered that although the tumor response to treatment was similar in TLR9 siRNA and control siRNA TNBC groups, mice bearing TLR9 siRNA tumors lost significantly less weight than similarly treated mice with control siRNA tumors. Similar weights of the vehicle-treated mice suggested to us that TLR9 expression in the tumors may be an important determinant of chemotherapy-induced inflammation and activation of anti-tumor immunity (47). Inflammatory response to chemotherapy is gaining acceptance as an important mediator of treatment responses to standard cancer therapy (63). More specifically, we hypothesize that the tumor TLR9-dependent, post-treatment weight loss is actually a surrogate marker for self-DNA-induced and TLR9-mediated inflammation that takes place at the tumor site. Such tumor TLR9-mediated inflammation might then amplify the anti-tumor immune response, eradicate microscopic disease and through this mechanism, translate into cure (47). We predict that the lack of such immunogenic effect in tumors that have low-TLR9 expression indeed contributes to the described poor disease-specific survival in triple-negative disease (32). This hypothesis requires a detailed analysis of tumor TLR9-dependent immune response to chemotherapy in immune-competent pre-clinical cancer models. However, if true, it would mean that patients with low-TLR9–TNBC could especially benefit from adjuvant cancer immunotherapy. It is also possible that TLR9 expression changes tumor immunophenotype independent of treatment and this aspect also requires further investigation.

## TLR9 Regulation in Breast Cancer

Several cancer-associated viruses have been shown to down-regulate TLR9 expression through their oncoproteins. For example, human papillomavirus (HPV), Epstein–Barr virus, and hepatitis B virus inhibit the expression and impair the function of TLR9 in infected target cells (64–66). Patients with chronic hepatitis B virus have decreased levels of TLR9 mRNA in peripheral blood mononuclear cells (67). The Merkel cell polyomavirus large T antigen down-regulates TLR9 expression in epithelial cells and in cells derived from Merkel cell carcinomas (68). For the HPV16, the mechanism behind TLR9 suppression was recently shown to involve the viral oncoprotein E7-induced formation of transcriptional inhibitory complex that includes NF-κB p50–p65, ERα, and chromatin modifying enzymes. This complex induces epigenetic changes at the TLR9 promoter area (69). It is likely that these viral effects on TLR9 expression and function play an important role in viral persistence, through inhibition of host immune responses (64, 65, 67, 70, 71). Nevertheless, also opposite effects on microbial TLR9 regulation have been suggested (72, 73). Although breast cancer is not currently considered to have viral etiology, several viruses, including human papilloma viruses, have been detected in normal and cancerous human breast tissues (74–77). Whether or not these viral effects have a role in breast cancer development or pathophysiology is currently unknown.

Tumor microenvironment oxygen concentration is also an important regulator of TLRs. Similar with the effects of hypoxia on other TLRs in other cell types, hypoxia also up-regulates TLR9 expression in breast cancer cells *in vitro* and in orthotopic breast tumors *in vivo* (32, 51, 78). These hypoxia effects on TLR9 mRNA and protein expression were mediated via HIF-1α in breast cancer cells *in vitro* (32). TNBCs are typically hypoxic (79). Therefore, understanding the mechanism on why tumor TLR9 expression levels remain low despite hypoxia in some TNBCs might open novel therapeutic possibilities that might also apply to renal cell carcinoma (30). It was also demonstrated recently that TLR9 expression is under the control of the circadian molecular clock (80). The significance of this finding for breast and other cancers is currently open.

Although TLR9 is expressed in all clinically relevant subtypes of breast cancer, we and others have discovered that there is an inverse correlation between tumor TLR9 and ER expression: ER-positive breast cancers have significantly lower levels of TLR9 expression, as compared with TNBCs (26, 29, 32, 36). The basal TLR9 expression is also significantly lower in human ER-positive breast cancer cells, as compared with human ER-negative breast cancer cell lines *in vitro*. Furthermore, transfection of ERα cDNA into TNBC cells suppresses TLR9 expression of the recipient cells (36). Both estradiol and testosterone induced TLR9 expression via their cognate receptors in breast cancer cells *in vitro*. Testosterone also augmented the pro-invasive effects of CpG–ODNs. Finally, bicalutamide, a commonly used hormonal treatment in prostate cancer, increased TLR9 expression in ER-positive breast cancer cells (36). This effect of bicalutamide on TLR9 expression might be of therapeutic interest since a proportion of TNBC tumors express the androgen receptor that bicalutamide targets (81).

## TLR9 Polymorphism in Breast Cancer

The TLR9 gene is located on human chromosome 3 (82). Although TLR9 gene polymorphisms have been studied in other diseases, including infectious and autoimmune diseases and some cancers, very little is known about TLR9 gene polymorphism in breast cancer (83–86). A study conducted by Resler and coworkers using over 800 case and control samples, found that the single nucleotide polymorphism (SNP, rs352140) in *TLR9*, which does not alter protein amino acid sequence but might alter protein function or stability, was associated with breast cancer risk (OR 0.85, 95% CI 0.74–0.97) (87). The patients in this study were all post-menopausal (65–79 years) and 80% of the cases had hormone receptor-positive breast cancer. These results were in contrast to those of Etokebe et al., who found no association in the same TLR9 SNP with breast cancer risk in a small Croatian cohort, consisting of 130 breast cancer cases and 101 controls (88).

## Conclusion

Although TLR9 is widely expressed in breast cancers, it appears that tumor TLR9 expression has prognostic significance only in TNBC. Especially, TNBC patients that have low tumor TLR9 expression upon diagnosis have a significantly shortened disease-specific survival, as compared with TNBC patients that have high tumor TLR9 expression. These findings, however, need to be repeated in larger and more diverse patient populations. TNBC tumors are typically hypoxic and low oxygen concentrations up-regulate TLR9 expression in TNBC cells in pre-clinical models. Understanding why TLR9 expression levels remain low in some TNBC tumors in the hypoxic tumor microenvironment might reveal novel therapeutic opportunities. It has been demonstrated recently that viral oncoproteins down-regulate TLR9 expression in various cancer tissues. Although breast cancer is not currently considered to have viral etiology, various viruses known to be capable of down-regulating TLR9 expression have also been detected in breast cancers. The contribution of these viral infections to low tumor TLR9 status in TNBC should therefore be addressed in future studies. Finally, the mechanisms how the lack of tumor TLR9 expression results in poor prognosis are unknown. Studies from pre-clinical TNBC models suggest that tumor TLR9 expression might affect tumor immunophenotype or be required for chemotherapy-induced anti-tumor immune response. If this is the case, then patients with low-TLR9–TNBC tumors might benefit from anti-cancer immune therapy. The specificity of the immune therapy requires, however, a clear understanding of how TLR9 expression affects tumor immunity. Synthetic TLR9 agonists, CpG–ODNs have demonstrated promising direct and immune system-mediated anti-cancer effects against breast cancer in pre-clinical models but they have not been studied in clinical breast cancer trials. It is clear that synthetic CpG–ODNs induce cancer-cell invasion *in vitro*. Whether this finding is relevant for the clinical situation, where such agonists are given in order to boost the anti-tumor immune response, remains to be resolved. Finally, aiming to increase tumor TLR9 expression prior to chemotherapy should be considered a therapeutic opportunity in the TNBC patients that have low tumor TLR9.

## Conflict of Interest Statement

The authors declare that the research was conducted in the absence of any commercial or financial relationships that could be construed as a potential conflict of interest.
